# Tightrope act: autophagy in stem cell renewal, differentiation, proliferation, and aging

**DOI:** 10.1007/s00018-012-1032-3

**Published:** 2012-06-05

**Authors:** Kanchan Phadwal, Alexander Scarth Watson, Anna Katharina Simon

**Affiliations:** 1 BRC Translational Immunology Lab, NIHR, Nuffield Department of Medicine, John Radcliffe Hospital, University of Oxford, Oxford, OX3 9DU UK; 2 MRC Human Immunology Unit, Weatherall Institute of Molecular Medicine, John Radcliffe Hospital, University of Oxford, Oxford, OX3 9DS UK

**Keywords:** Autophagy, Stem cells, Lysosomes, Senescence, Aging, HSC, ESC, MSC, NSC

## Abstract

Autophagy is a constitutive lysosomal catabolic pathway that degrades damaged organelles and protein aggregates. Stem cells are characterized by self-renewal, pluripotency, and quiescence; their long life span, limited capacity to dilute cellular waste and spent organelles due to quiescence, along with their requirement for remodeling in order to differentiate, all suggest that they require autophagy more than other cell types. Here, we review the current literature on the role of autophagy in embryonic and adult stem cells, including hematopoietic, mesenchymal, and neuronal stem cells, highlighting the diverse and contrasting roles autophagy plays in their biology. Furthermore, we review the few studies on stem cells, lysosomal activity, and autophagy. Novel techniques to detect autophagy in primary cells are required to study autophagy in different stem cell types. These will help to elucidate the importance of autophagy in stem cells during transplantation, a promising therapeutic approach for many diseases.

## Introduction

### Autophagy

Macroautophagy (hereafter referred to as autophagy) is a bulk lysosomal degradation pathway involved in recycling long-lived proteins and cytoplasmic organelles [1]. It is negatively regulated by serine/threonine kinase mTOR (mammalian target of rapamycin) and is a key process for cellular growth and metabolism [2–5], with its end products generating macromolecules to maintain cellular homeostasis. The process starts with the formation of double-membraned autophagosomes, which sequester cargo and fuse with acidic lysosomes to form autolysosomes, wherein the cargo is degraded by acid hydrolases to release the end products. There are over 30 autophagy-related (Atg) genes identified so far and these genes are highly conserved among eukaryotes. Atg8 and Atg12 encode two ubiquitin-like proteins; Atg12 is conjugated to Atg5 by Atg7 and Atg10, E1 and E2-like proteins, respectively, while Atg7 and Atg3 act to conjugate Atg8/LC3-I to phosphatidylethanolamine (LC3-IPE/LC3-II) [6]. Finally, Atg12–Atg5 and Atg8 localize to developing autophagosomes, which after completion fuse with lysosomes. On induction of autophagy, the conversion of LC3-I into LC3-II is indicative of autophagosome formation, thus is widely used as a marker for autophagosome formation. This pathway depends heavily on lysosomal activity and any defect in lysosomal degradation of autophagosomes can lead to their accumulation, impairing cell function and possibly leading to cell death by caspase activation [7]. As such, lysosomal inhibitors impair autophagic degradation; examples of these along with other pharmacological and genetic modulators are presented in Table 1. Originally viewed as a non-selective, bulk process, it is now known that specific proteins and organelles are selected and degraded by autophagy (selective autophagy) via LC3-interacting adaptors such as p62 and NBR1 [8] and autophagy receptors like Nix [9].

**Table 1:** List of autophagy modulators in stem cells

	Mode of action	References
Modulator		
mTOR	mTOR inhibition induces autophagy	[13, 46, 85, 127, 88]
miR-17, 20, 93, 106	Targets SQSTM1/p62	[51–53]
PTEN	A tumor suppressor, autophagy inducer via AKT	[92, 128, 94, 118–120]
P53	A tumor suppressor, inhibition of p53 induces autophagy	[30, 128, 65, 31, 83, 29]
DRAM	Stress-induced regulator of autophagy	[129]
HDAC6	Histone deacetylase-6, quality control of autophagy, regulates autophagosome-lysosomal fusion	[108]
TFEB	Transcription factor EB, master transcription factor for lysosomal biogenesis, drives expression of autophagy and lysosomal genes	[109]
mTORC1	Phosphorylates the ULK1-mAtg13-FIP200 autophagy regulatory complex, inhibits autophagy	[39, 107]
Chemical modulators		
Starvation and dietary restriction (DR)	Autophagy inducer via mTOR inhibition/SIRT-dependent pathway	[10, 101]
Rapamycin	Autophagy inducer via mTOR inhibition	[13, 113, 115, 116]
Chloroquine (CQ)	Lysosomotropic agent, inhibits fusion of lysosomes with autophagosomes	[7, 49]
3-methyladenine (3MA)	Autophagy sequestration inhibitor	[13, 115, 116]
LY294002	Inhibits autophagy via phosphoinositide 3-kinases	[13]
Bafilomycin A (BafA)	Vacuolar ATPase inhibitor, interferes with the autophagosome–lysosomal fusion step	[7]
E64D/pepstatin A (PepA)	Inhibition of lysosomal hydrolases, blocks the flux of autophagic pathway	[113]

**Fig. 1:**
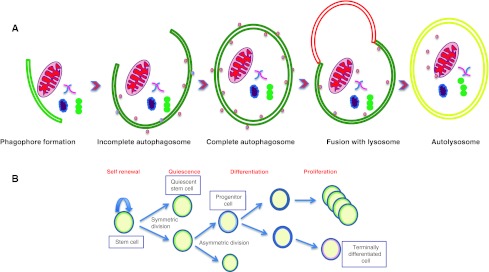
**a** Autophagy, a lysosomal degradation pathway engulfing organelles and protein aggregates. **b** Autophagy is involved in maintaining and regulating all the basic properties of stem cells like self-renewal, quiescence, differentiation, and proliferation

Several studies implicate autophagy in the cellular response to stressors, including damaged mitochondria, protein aggregation, tumors, hypoxia, aging, bacteria and viral infections, therapeutic stress and nutrient starvation [10]. Moreover, as an intracellular recycling pathway autophagy plays a crucial role in the active elimination of unnecessary proteins and organelles, such as those that accumulate during the dynamic programme of stem cell renewal, proliferation and differentiation [11–13] (Fig. 1). It not only provides bioenergetics support for these events but also takes care of apoptotic bodies generated during the process.

### Stem cells

Stem cells are non-specialized cells capable of differentiating into many different types of mature cells while maintaining the stem cell population through self-replication. Stem cells are long-lived and undergo lengthy periods of quiescence. We hypothesize that these unique properties of stem cells, namely (1) pluripotency and differentiation, (2) self-renewal, and (3) quiescence require autophagy (Fig. 1):

(1 and 2) The processes of differentiation and self-renewal require a strict control of protein turnover and lysosomal digestion of organelles. [13]. Autophagy, as a highly inducible catabolic process, plays a pivotal role in the attainment of precise morphology and function by controlling protein turnover via mTOR regulation. Taking cues from the environment and the hormones present, autophagy specializes in the task of ‘remodeling’ stem cells. A fast and efficient removal of sets of transcription factors, enzymes, adhesion molecules, or released factors can be efficiently accomplished by autophagy, this being a great advantage for both differentiation and renewal. (3) Moreover, stem cells spend most of their life in the reversibly arrested cell cycle phase (G_0_ phase) waiting for cues to re-enter the cell cycle and differentiate into progenitors and tissue-specific cells [14]. Autophagic turnover of proteins and fatty acids within these quiescent cells presumably plays a significant role in eliminating damaged macromolecules, which could cause loss of quiescence and cannot be diluted by transmission to progeny cells [15]. For example, mitophagy is essential to preserve the ‘stemness’ or pluripotency of embryonic stem cells within in vitro long-term cultures by resisting genetic and/or mitochondrial damage [16].

Depending on their location in the body and the type of cells they produce, stem cells can be classified as embryonic stem cells (ESCs) and adult stem cells (ASCs). ASCs include hematopoietic (HSCs), mesenchymal (MSCs) and neuronal stem cells (NSCs). Here we briefly review the role of autophagy in stem cell renewal, differentiation, development and aging (summarized in Table 2), highlighting some most recent methods available to study autophagy in these rare populations. Finally we will address how it can be modulated for stem cell-based gene therapy and other stem cell targeting approaches.

**Table 2:** Role of autophagy in development, differentiation, and aging of stem cells

Type of stem cells	Role	References
Embryonic stem cells	Clearance of dead cells during embryonic morphogenesis, efficient removal of apoptotic cells, removal of defective mitochondria and ubiquitinated proteins, maintain proliferation, degrades midbodies in ESC differentiation	[16, 26, 28, 33, 35, 36]
Adult stem cells	Cellular remodeling during differentiation and development, maintain quiescence	[37, 38]
Hematopoietic stem cells	Homeostatic proliferation, effective mitochondrial removal, preventing DNA damage, self-renewal, lineage fate	[40, 41, 43–45, 49, 52]
Mesenchymal stem cells	Cytoprotective response, differentiation	[58, 63]
Neuronal stem cells	Differentiation, survival under nutrient stress	[13]
Stem cell aging	HSC proliferation and function, stem cell senescence, immunosenescence, myelogenesis, removal of defective mitochondria, proteolysis, reducing oncogenesis	[85–88, 94, 101, 102]

### Detection of autophagy in rare cell populations

Stem cells are a very rare population within tissues, making them difficult to identify and therefore difficult to study. Much effort has been made in recent years to find molecular markers that identify these populations in different tissues types [17–19, 20]. Being small populations with a combination of multiple markers required to define them, stem cells call for more modern techniques in order to study their autophagy levels. Existing standard methods of autophagy detection include western blots (measuring the ratio between LC3-I and LC3-II), electron microscopy or immunofluorescence detection of LC3 (dispersed to punctate localization) or lysosomal methods using LAMP1/lysotracker (on their own or colocalized). However, it is worth noting these techniques may not necessarily give an accurate quantification of autophagy in the absence of lysosomal inhibitors, thus some of the data based on these readouts may need confirmation in the presence of such inhibitors to get an accurate picture of autophagy levels (Klionsky 2011 Guidelines for the use and interpretation of assays for monitoring autophagy in higher eukaryotes). In order to use any of the available traditional autophagy detection techniques [21] on these rare primary stem cell populations, cells need to be sorted; however, both flow cytometry or bead sorting stresses cells, thereby inducing autophagy and reducing the signal to background ratio. Although there are a few flow-cytometry-based methods available, these have mostly been applied to LC3-GFP transfected cells [22, 23] rather than primary populations.

Recent advances have begun to allow the study of autophagy in stem cell populations. Tra et al. have generated human embryonic stem cell lines stably expressing GFP-LC3. These cells lines, known as HES3-GFP-LC3, express both GFP and the pluripotency marker TG30 along with markers like Oct4, TRA-181 and TRA-160, typical characteristics of undifferentiated hESCs. When injected into SCID mice, they differentiate into mesoderm, endoderm and ectoderm layers. This could prove to be a very useful tool to investigate autophagy in hESCs and hESC derived cell populations [24]. Furthermore for the detection of autophagy in primary murine stem cells, transgenic mice expressing LC3-GFP ubiquitously can be used. However, to study autophagy levels in human stem cells, and while avoiding the stressful and possibly autophagy-inducing xenografting, our lab has recently developed a novel image-cytometry-based high-throughput assay for autophagy measurement in primary cell populations [25]. This assay combines surface markers with autophagy detection. Endogenous LC3 is detected by anti-LC3 antibody and lysosomal content by lysosomal markers, and the quantification of their colocalization gives an accurate measure of autophagy levels. This unique technique, combined with lysosomal inhibitors or evidence of subsequent degradation and recycling of cargo, could give insight into autophagic flux in these rare human stem cell populations without subjecting them to any sorting procedure.

## Autophagy in stem cell compartments: live and let live

### Embryonic stem cells (ESCs)

ESCs are pluripotent stem cells present in the early embryo with the capacity to differentiate into the primary germ layers: ectoderm, endoderm, and mesoderm. The role of Atg5/Apg5 in ES cell development was first demonstrated using null mutation of the Atg5 gene in murine ES cells [26], with the cells showing impaired autophagosome formation by electron microscopy, suggesting a block in the autophagy pathway, and reduced bulk protein degradation. During the autophagic process, Atg5 is conjugated to Atg12 to form an ubiquitin protein ligase (E3) like enzyme for Atg8-PE conjugation [27]. Beclin 1, mammalian homologue of yeast Atg6/Vps30, is a component of the class III phosphatidylinositol-3-OH kinase [PI(3)K] complex. Studies on beclin1^−/−^ deficient embryonic stem cells suggest a critical role for autophagy in maintenance of tissue homeostasis in vivo, as mice generated from these ES cells die early in embryogenesis [28]. In the absence of autophagy, beclin1^−/−^ embryonic stem cells undergo p53-dependent apoptotic cell death involving Bcl-2 family proteins and caspase activation on exposure to UV light [29–31], and a similar fate awaits them on serum deprivation but in a caspase-independent manner [32]. Moreover, both Atg5^−/−^ and beclin1^−/−^ murine ES cells show a defect in embryoid cavitation during embryonic morphogenesis; Atg5 and beclin1 are not only essential for the clearance of dead corpses during mammalian embryonic morphogenesis but also for initiation of “eat-me” signals required for efficient removal of apoptotic cells [33]. Pluripotent ESCs have a small number of mitochondria and their maintenance relies on efficient mitophagy. Defect in key gene Gfer (the mammalian homologue of yeast erv1), which is involved in the mitophagy pathway, leads to the loss of structural and functional integrity of ESC mitochondria, affecting ESC pluripotency and survival [34]. Finally, homozygous mutation of the autophagy gene Ambra1 also causes embryonic lethality in mice and its functional deficiency in embryonic stem cells leads to severe neural tube defects and accumulation of ubiquitinated proteins in cells, all linked to autophagy impairment and dysregulation. Moreover, lack of Ambra1 during embryogenesis leads to over proliferation and increased apoptosis in the neuroepithelium [35], emphasizing the role of Ambra1 in controlling cell proliferation and cell survival during nervous system development.

More evidence of autophagy’s role in hESC differentiation comes from HES3-GFP-LC3 cell lines. During their differentiation both increased LC3 punctae and increased accumulation of LC3-II were observed, while further acute induction of hESC differentiation with TGF β receptor II inhibitor led to a rapid increase in GFP-LC3 punctae. As no increase in GFP-LC3 punctae was observed later in differentiated hESCs, this suggests a strong connection between early differentiation events and autophagy induction [24]. However, this inhibitor also led to mTOR inhibition in hESCs, thus study of hESC differentiation induced by small molecules that do not inhibit mTOR will be necessary for deciphering and establishing the role of autophagy in these stem cells. In general, mTOR regulates cell growth and metabolism in response to nutrients, growth factors, cellular energy, through modulating translation, transcription, ribosome biogenesis, nutrient transport, and autophagy. It is thought that the integrated outcome of mTOR signaling leads to a coordinated cellular program, of which autophagy is an integral part.

Interestingly though, a recent paper suggests that evasion of autophagic degradation and/or low autophagy levels may be required for embryonic stem cell maintenance by allowing accumulation of midbodies. Midbodies, organelles formed during cell division and inherited asymmetrically by daughter cells, can be degraded through autophagy/lysosomal pathways but were shown to accumulate in ESCs and induced pluripotent stem cells, with enrichment in the latter improving stem cell reprogramming efficiency [36]. Thus, as will be a theme throughout this review, it seems a controlled balance of autophagic activity may be vital for ESC maintenance.

### Adult stem cells: HSCs

Adult stem cells (ASCs) are self-renewing, multipotent cells found throughout the body; they can divide to give rise to another stem cell or a progenitor/precursor cell (hematopoietic, mesenchymal and neural), which can further differentiate into specialized cell types. They are rare and their main function is to maintain cellular homeostasis. Several labs have suggested a role for autophagy in cellular remodeling during differentiation and development of different ASCs [37, 38], with the majority of data available on its role in hematopoietic stem cells (HSCs).

A study on mice lacking ATM (ataxia-telangiectasia mutated kinase), a molecule that is indirectly involved in autophagy and has a direct role in DNA damage response, shows a loss of function in HSC self-renewal. Located in the cytoplasm, ATM acts as a sensor of ROS and can signal via LKB1, AMPK, and TSC2 in the cytoplasm to deactivate mTORC1, hence inducing autophagy [39]. HSCs from ATM^−/−^ mice were profoundly depleted, with those remaining showing high levels of reactive oxygen species (ROS), very similar to Atg7^−/−^ HSCs [40]. They also show increased levels of tumor suppressors proteins including p16^INK4a^ and p19^ARF^ [41]. HSCs were restored by NAC (*N*-acetyl-l-cysteine) or catalase treatment, which acted as anti-oxidants and also down regulated p16^INK4a^ and p19^ARF^. It is possible that inducing autophagy in ATM^−/−^ HSCs may have had a similar effect on HSC restoration, as autophagy has a significant role in the ROS response (a circuit possibly interrupted by ATM mutation) and removal of ROS-damaged organelles. This could be tested in future studies along with use of small molecule inhibitors for ATM and related signaling molecules LKB1, AMPK and TSC2. Interestingly ATM also regulates centrosome biogenesis and thereby suppresses genome instability and cellular transformation [42]. Midbodies along with older centrosomes are inherited asymmetrically by the daughter cell and selectively accumulate in stem cells [42]. It is possible that centrosomes get degraded along with midbodies in stem cells by autophagy and that ATM plays a role here.

Similarly, the differentiation of HSCs into mature white and red blood cells and the maintenance of hematopoietic lineages are affected by the loss of autophagy. A hematopoietic chimeric mouse generated with Atg5^−/−^ fetal liver results in T lymphopenia due to enhanced CD8^+^ T cell apoptosis and an inability to undergo TCR-induced proliferation, suggesting a crucial role of autophagy in T cell survival and function. These mice also show reduced numbers of thymocytes and B lymphocytes, suggesting Atg5’s importance in homeostatic proliferation of lymphocytes [43]. Likewise, studies from our lab have demonstrated that the absence of Atg7 in the hematopoietic system (tissue-specific excision with Vav-Cre) leads to defective removal of mitochondria in erythroid cells, with the mice developing severe progressive anemia [44].

Furthermore Atg7 has a non-redundant role in hematopoietic stem cell function and myeloid proliferation [40]. The autophagy deficient hematopoietic stem and progenitor cells (HSPCs) from Atg7^−/−^ mice were unable to form secondary colonies during colony-forming cell (CFC) assays, suggesting a defect in self-renewal. The lethally irradiated recipients of Lin^−^Sca^+^c-Kit^+^ (LSK) cells, containing the HSPCs, purified from Vav-Atg7^−/−^ mice died within weeks of transplantation during noncompetitive repopulation assays, suggesting that autophagy is indispensable for normal HSC function. In line with Atg5^−/−^ chimeras, these mice had significantly reduced lymphoid progenitor compartments in the bone marrow (BM) and lymphopenia. Although the mice showed an early overall expansion of the Vav-Atg7^−/−^ LSK compartment, the true HSCs (LSKCD150^+^CD48^−^) were significantly reduced. Moreover, the expanded LSK compartment contained increased levels of mitochondria, suggesting impaired mitophagy, which leads to enhanced ROS production and DNA damage triggering both apoptosis and proliferation. The mice developed myeloproliferation and died of BM failure by 12 weeks. We concluded that autophagy is essential for self-renewal, proliferation and proper functioning of HSCs. Similarly, a hematopoietic and endothelial-specific knock out mouse of FIP200 (focal adhesion kinase family-interacting protein of 200 kD) under the Tie-2 promotor showed a very similar phenotype of HSC depletion, loss of HSC reconstitution capacity, a block in erythroid maturation and aberrant expansion of myeloid cells with increased mitochondrial load and DNA damage [45]. FIP200 is an essential autophagy gene and the mammalian counterpart of yeast Atg17, part of the ULK-Atg13-FIP200 complex, which regulates autophagy in mTOR controlled manner [46]. Interestingly, Tie2-FIP200^−/−^ mice die in utero or shortly after birth. This early lethality observed in the Tie-2-driven model as opposed to the Vav-driven excision of Atg7 may be explained by either (1) the fact that Tie-2 also drives excision in endothelial cells thereby damaging an endothelial niche indispensable for HSC development or by (2) the different requirement/roles of FIP200 and Atg7 in the autophagic process.

To preserve long-term regenerative capacity and maintain resistance to acute physiological stress, stem cells modulate between quiescent and proliferative states. LKB1 (a tumor suppressor, Serine/threonine kinase 11) has a role in maintaining cellular energy levels (ATP/AMP ratio), phosphorylating AMPK in response to a decline in the cellular ATP/AMP ratio [47, 48]. When LKB1 is knocked out in murine HSCs, increased cell death and LC3II levels were observed in bone marrow, thymus and spleen [49]. While levels of LC3II were not measured in the presence of lysosomal inhibitors, making interpretation of these results problematic, treatment with chloroquine, an autophagy inhibitor, accelerated the death of LKB1 mutants suggesting that autophagy helps to rescue HSCs in the absence of LKB1. Autophagy may therefore act as a compensatory mechanism during metabolic stress in LKB1 mutant HSCs.

Recent data using expression profiling suggests that HSC commitment, proliferation, survival, and differentiation are regulated by miRNAs [50, 51]. It has been shown that miR-17, -20, -93 and -106 are expressed abundantly in mouse and human hematopoietic progenitor cells and their expression considerably declines during myeloid differentiation [52] with a proven role in myeloid development [51, 53]. Quantitative proteomic studies revealed that these miRNAs target and downregulate Sequestome 1 (SQSMT1). The SQSTM1 gene encodes p62, a multifunctional signal adaptor scaffold protein, which regulates the transport and targeting of poly-ubiquitinated proteins destined for degradation by autophagy and proteasomal pathways [54–56]. These modified proteins predominantly colocalize with late endosomes and lysosomal markers RAB7 and LAMP1 [57], thus p62-dependent degradation via lysosomes may have a significant role to play in myeloid cell expansion/regulation [52]. However, these results are in contrast with studies showing myeloid expansion in the absence of key autophagy genes FIP200 or Atg7 [44, 45], suggesting either a specific quantitative or temporal restriction to autophagy’s requirement during certain stages of myeloid development.

### Adult stem cells: MSCs

MSCs are multipotent stem cells that can differentiate into a variety of cell types, including osteoblasts (bone cells), chondrocytes (cartilage cells), adipocytes (fat cells), fibroblasts and endothelial cells. A role for autophagy protein beclin1 in MSCs was first shown in a novel pathway-involving ERK-mediated induction of cyclin E via beclin1 [11]. It is known that cAMP promotes differentiation of mesenchymal stem cells into neuron-like cells [58] and the inhibition (via U0126 and siRNA) of ERK kinase MEK significantly reduced cAMP-mediated autophagy. Activation of ERK has been associated both with induction or inhibition of autophagy depending on the cell types studied [59–62]. Possibly these results suggest a role for autophagy, via cAMP induction in MSC differentiation and in protection against neurological disorders. Moreover, with indications that autophagy activation in MSCs helps to support the microenvironment of developing solid tumors [63], understanding the roles of autophagy in MSCs may have therapeutic value.

Furthermore, loss of Atg7 and 5 in a preadipocytic cell line resulted in reduced triglyceride accumulation, along with reduced induction of adipocyte differentiation markers. Pharmacological inhibition of autophagy with 3-MA also showed a similar trend, although to a reduced extent. Interestingly, nonspecific lysosomal inhibition with ammonium chloride and leupeptin resulted in an analogous phenotype, demonstrating the critical role of the autophagosome-lysosomal pathway in adipocyte differentiation [37].

### Adult stem cells: NSCs

A potential role for autophagy during in vitro neuronal differentiation was demonstrated in mouse neuroblastoma N2a cells, where retinoic acid-induced neuronal differentiation showed a time-dependent increase in the LC3II/LC3I ratio. GFP-LC3 punctae were localized to the soma and neuritis of the differentiated cells. Moreover, chemical inhibition of autophagy using 3-methyladenine (3-MA) and LY294002 and genetic inhibition via siRNA knock down of beclin1 resulted in differentiation defects/delays. Intriguingly, addition of rapamycin impaired cell differentiation as well, indicating that perhaps neuronal differentiation, similar to that of ESCs and HSCs above, depends on a very delicate balance of autophagic activity in these cells, as neither a complete inhibition nor induction (inhibition of mTOR) of autophagy allows proper differentiation of NSCs [13]. However, impact of rapamycin on autophagy-unrelated pathways may also play a role.

Normal lysosomal function is essential for the proper functioning of the autophagy machinery and any defect in lysosomal function can lead to accumulation of autophagosomes. Lysosomal dysfunction has been shown to cause death in neuronal and non-neuronal cells [64]. Neural or neuronal precursor cells (NPCs) have high expression of p53 (a tumor suppressor) and p53 has a crucial role in both the autophagy and apoptosis pathways [65]. Using the lysosomal inhibitors chloroquine (CQ) and bafilomycin A1 (BafA1, a vacuolar ATPase inhibitor) in NPCs or a neural stem cell line resulted in a time-dependent aberrant accumulation of autophagosomes, p53 phosphorylation, increased damage-regulatory autophagy modulator levels (DRAM), and caspase 3-dependent cell death. shRNA knockdown of Atg7 significantly attenuated CQ and BafA1-induced cell death [7]. This is in contrast to most other studies that clearly show a pro-survival role for Atg7-mediated autophagy and it is not clear why CQ treated cells survive better in the absence of Atg7. Furthermore recent in vitro studies have shown increased autophagy levels (Atg7, Becn1, LC3 and Ambra1) in mouse embryo olfactory bulb stem cells during early stages of neuronal differentiation. Ambra1 haploinsufficiency and ATG5^−/−^ resulted in reduced neuronal differentiation both in vivo and in in vitro cultures. Inhibition of autophagy using 3-MA and Wortmannin reduced neuronal differentiation. Interestingly, adding a citric acid cycle analogue methyl pyruvate reversed the effect [66]. This study indicates a homeostatic role for autophagy as an energy provider during the early stages of neuronal differentiation.

Along these lines, a recent study has reported increased levels of autophagy (punctate immunostaining patterns for LC3 and Atg5 along with increased conversion of LC3-I to LC3-II and reduced p62 levels) in epidermal (ESC), dermal (DSC), and HSC when compared to control primary immature keratinocytes, fibroblasts and neutrophils, suggesting upregulation of autophagy in these stem cells. These three stem cell types lost their capacity to self-renew (capacity to form colonies) and differentiate when treated with 3-MA for 24 h or when transfected with ATG5 shRNA, hence losing their ‘stemness’. Furthermore, when autophagy was blocked, the stem cells underwent significant etoposide-induced apoptotic cell death [67]. Interestingly, autophagy not only plays a significant role in self-renewal and differentiation of these adult stem cells, it also aids their survival under hostile cytotoxic environment of damage and dysfunction.

**Fig. 2:**
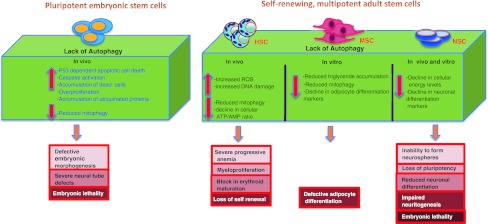
A summary model depicting implication of loss of autophagy, the symptoms and consequences observed in various in vivo and in vitro studies in ESCs and ASCs

These data in HSCs, MSCs, DSCs, and NSCs, together suggest a vital role for the autophagic program in adult stem cell function; a list of all the systemic knockout mice of ATG related genes and their respective stem cell phenotypes can be found in Table 3 and a schematic summary in Fig. 2.

**Table 3:** Phenotypes of knockout mice of autophagy-related genes in stem cells

Genes^genotype^	Tissue/promoter	Phenotype	References
Beclin1^−/−^	ESC	Die early in embryogenesis	[28, 33]
Beclin^+/−^	ESC	Develop tumors, apoptotic cell death	[28]
Atg5^−/−^	ESC	Autophagy-dependent defective clearance of apoptotic cell corpses during development	[26, 43]
Ambra1^−/−^	ESC	Embryonic lethality	[35]
Ambra^gt/gt^	ESC/gene-trapped allele	Over proliferation, increased apoptosis	[35]
Atg7^fl/fl^	HSC/Vav	Unable to self-renew, defective mitochondrial removal, significantly reduced myeloid and lymphoid progenitors in BM, myeloproliferation of LSK compartment, myeloid leukemia	[12, 40, 44]
FIP200^fl/fl^	HSC/ Tie-2	Embryonic lethality, HSC depletion, loss of self-renewal, block of erythroid maturation, myeloproliferation, increased mitochondrial load and DNA damage	[45]

### Selective autophagy in stem cell reprogramming and differentiation

Studies on autophagy receptors and adaptors involved in selective autophagy of protein aggregates (aggregophagy) and organelles (mitophagy for mitochondria, reticulophagy for ER and ribophagy for ribosomes) in stem cells are very few but nonetheless are interesting and intriguing. Nix (also known as BNIP3L) binds to LC3/GABARAP via LIR-W35 and has a carboxyl terminal domain essential for targeting the proteins to the outer mitochondrial membrane [9]; recent models have Nix and BNIP3 itself acting as a tether between mitochondria and the forming autophagosome [68]. Nix^−/−^ mice generated using ES cells showed low RBC count, polychromasia, absence of early erythroblasts and increased reticulocytes, all pointing towards defects in erythrocyte maturation. Removal of mitochondria is key to erythrocyte maturation but Nix^−/−^ reticulocytes retained mitochondria both in CD71^+^ and CD71^−^ RBCs [69]. Although a few of the mitochondria were observed inside autophagosomes, the majority were lying outside, indicating a potential defect in targeting these mitochondria to autophagosomes; ribophagy, however, was observed to be normal. Along with reduced mitophagy, Nix^−/−^ RBCs had a shorter life span, increased ROS and spontaneous caspase activation in in vitro cultures.

Furthermore, autophagy adapters like NBR1 are involved in selective degradation of midbody derivatives (MB^d^), which, as mentioned above, accumulate in stem cells and are lost during their differentiation. Cells stably expressing NBR1-specific shRNAs showed increased MB^d^ accumulation over control cells and have increased reprogramming efficiency (increased iPSC colony formation) along with enhanced pluripotency [36]. Another study described the role of the p62 adaptor in neuronal differentiation. HiB5 cells (a multipotent hippocampal stem cell line) can differentiate into neuronal cells and enhanced expression of p62 was observed in efficiently differentiating HiB5 cells. Over-expression of p62 protein in HiB5 cells resulted in increased neuronal differentiation markers like neurofilaments and synapsin Ia. Interestingly, p62 overexpression also increased cell survival under serum starvation [70]. A more recent study shows p62-based selective autophagy is involved in myeloid cell line differentiation via degrading PML–RARα [a fusion protein of promyelocytic leukemia (PML) and the retinoic acid receptor-α (RARα)], while inhibition of autophagy by shRNA targeting essential autophagy genes (ATG1 and 5) and pharmacological inhibition with 3-MA blocked both all-trans retinoic acid (ATRA)-induced PML–RARα degradation and consequently myeloid cell differentiation. Knockdown of p62 impaired this degradation and over expression of p62 increased the delivery of PML–RARα proteins to the lysosomes [71]. Another interesting autophagy cargo receptor is c-Cbl. Cbl family proteins are components of ubiquitin ligation machinery involved in degradation of phosphorylated proteins [72]. It has been hypothesized that they also mediate the ubiquitination and degradation of activated KIT. KIT is a tyrosine kinase (RTK) stem cell factor receptor and has an essential role in development of stem cells involved in hematopoiesis, pigmentation and reproduction [73]. Experimental evidences point towards the degradation of KIT via c-Cbl requiring proteasomal and/or lysosomal pathways as inhibition of lysosomal proteases suppressed the degradation of KIT and led to accumulation of its ubiquitinated form [74]. In general, more targeted studies are needed to define the autophagic role of autophagic receptors and adaptors in stem cell homeostasis.

## Autophagy and lysosomal function in stem cell aging: live or let die

Cellular senescence is characterized by cell cycle arrest, leading to irreversible loss of proliferative potential [75] accompanied by a decline in homeostatic and regenerative capacity of cells [76, 77]. Although pluripotent stem cells are long lived, a progressive decline in telomere length with age is reported in HSCs [78] and human MSCs are reported to be limited to only 40-50 divisions [79]. Senescence is best characterized in MSCs, with aging MSCs classified as (1) “deteriorating MSC”, marked by changes in quantity and quality, impaired self-renewal, restricted Hayflick limit and undergoing senescence including inactivated telomerase, (2) “persisting MSC”, which are less prone to senescence but subject to aging, and finally (3) “perennial MSCs” that escape aging indefinitely [80].

Various stress resistance and longevity related signaling pathways, such as FoxO3 [81], NF-κB [82], p53 [83] and SIRT1 [84], are effective regulators of autophagic degradation. While the mTOR pathway has emerged as a key regulator of growth and proliferation in stem cells [85], it is also involved in maintaining quiescence by repressing mitochondrial biogenesis and reactive oxygen species (ROS) [86]. Removal of damaged mitochondria via autophagy (mitophagy) reduces the oxidative burden, thus linking the mitochondrial free radical theory of aging with longevity [87].

Further evidence is accumulating that mTOR inhibition can slow down cellular senescence. Furthermore, impairing autophagy by conditional deletion of Tsc1 (tuberous sclerosis complex protein 1, deletion leads to constitutively active mTOR) leads to premature aging of HSCs in young adult mice [88] and ‘aging phenotype’ characteristics, including a relative decrease in lymphopoiesis, impaired capacity to reconstitute the hematopoietic system and increased expression of cellular senescence markers like CDK inhibitors p16 (Ink4a), p19 (Arf), and p21 (Cip1). Inhibition of mTOR by rapamycin restored HSC function, indeed rapamycin treated HSCs showed enhanced regenerative capacity, decreased expression of cellular senescence markers and significantly extended life span. The hematopoietic systems of both old mice and humans show a skewed ratio between myeloid and lymphoid progeny, as well as decreased numbers of memory B cells and naïve T cells, thus leading to immune senescence and vaccine failure [89–91]. Enhanced production of B lymphocytes and decreased myelogenesis could be achieved in aged mice after rapamycin treatment accompanied by a rejuvenated immune response to influenza virus. Although mTOR is a master switch implicated in a variety of cell growth aspects, thus inhibiting mTOR may have initiated autophagy-independent functions, this evidence is suggestive that autophagy is contributing significantly to the anti-aging effect. In line with this and as mentioned above, our lab has shown that mice deficient for the key autophagy molecule Atg7 have a significantly increased myeloid population, impaired mitophagy and loss of HSC reconstituting capacity, all hallmarks of aging [40, 44]. The common idiopathic anemia in elderly people could be partly explained by declining HSC function due to lack of autophagy in these cells.

A factor in controlling the potential lifespan of humans is increased risk of cancer as we grow old. Cellular senescence is generally thought to prevent tumorigenesis and this may be particularly important in stem cells. PTEN, which is upstream of mTOR, stimulates autophagy [92] and inhibits senescence. A complete loss of PTEN (tumor suppressor phosphatase and tensin homolog) elicits a pro-senescence response thereby countering tumorigenesis [93]. Moreover, several studies suggest that PTEN activity is inhibited by high levels of ROS, such as those found in aged/ autophagy-low cells [94]. PTEN loss induced cellular senescence (PICS) may be aided by the reduced autophagy levels. The latter result from increased PI3K signalling, which activates mTORC1. Therefore, it can be hypothesized that expression and/or activity levels of PTEN in aging stem cells may control their proliferation capacities.

Apart from PICS, oncogen-induced senescence (OIS) is another tumor suppressive mechanism. An induction of autophagy was observed when senescence was induced by retroviral-expression of oncogenic *ras* (H-rasV12). Ras+ senescent cells showed presence of increased autophagosomes (more LC3II by immunoblotting) both under basal conditions and in presence of Baf-A, when compared to quiescent IMR90 human diploid and embryonic skin fibroblast cells. Further study of autophagy level kinetics in OIS indicated that LC3II levels were upregulated during the ‘transition phase’ between an initial ‘mitotic phase’ to the later ‘senescence phase’ of OIS induction, where the LC3II levels progressively decreased [95]. Although our lab observed lower autophagy in senescent cell populations, the brief burst of autophagy seen during the ‘transition phase’ between the ‘mitotic’ and ‘senescence phase’ may be a cellular homeostatic mechanism, driving increased protein turnover and macromolecule availability to meet the demands of cells undergoing bursts of hyperproliferative signaling in response to mitogenic oncogene Ras; the autophagy levels fall as soon the cells enter the senescence phase.

Increased expression of *p16* ^ink4a^, a tumor suppressor protein, is a preprogrammed response decreasing proliferative capacities of stem cells to alleviate the increase in cancer risk with age [96–100], thus setting the paradigm of balancing senescence and cancer risk as mentioned above. Calorie restriction (CR) seems to break this paradigm and autophagy is required for dietary restriction-mediated life span extension [101]. Thus CR not only reduces the loss of stem cell proliferation with age in BALB/c mice, it also maintains low levels of *p16* ^ink4a^ [102] and reduces the risk of cancer along with promoting proliferation of stem cells in the elderly.

**Fig. 3:**
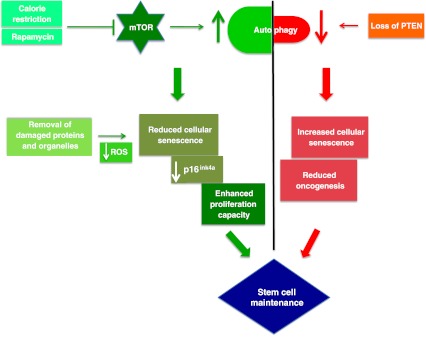
Tightrope act inhibition of mTOR via caloric restriction (CR) or rapamycin induces autophagy. Autophagy clears away damaged proteins and organelles like defective mitochondria, thereby decreasing ROS levels and reducing genomic damage and cellular senescence, thus playing a crucial role in enhancing stem cell longevity. CR may also have a role in maintaining low levels of p16ink4a, a tumor suppressor protein, thus reducing the risk of cancer and promoting proliferation of stem cells. Oncogenesis is countered by loss of PTEN which elicits a p53-dependent prosenescence response to decrease tumorigenesis

Figure 3 shows a schematic diagram of autophagy’s probable role in stem cell longevity.

### Lysosomes in stem cell aging

Age related increases in the oxidation of lipids and proteins within lysosomal membranes leads to increased volume and fragility of lysosomes, which may result in reduced fusion of lysosomes and autophagosomes and reduced levels of Lamp2a on lysosomal membranes. This in turn causes defective autophagy and chaperone-mediated autophagy [103]. In other ways, lysosomes are also a soft target of oxidative stress, with accumulation of the aging pigment lipofuscin and undigested materials in senescent cells [104]. Reduced autophagy or chaperone-mediated autophagy limits the proteolytic ability of senescent cells and hence limits adaptability to a changing environment, which could be very critical for a stem cell. As stem cell age and loose their proliferation potential, the efficiency of biological waste dilution by cell division also decreases. With age, stem cells show increased lipofuscin-related autofluorescence, carbonyl content and oxidative stress, as seen in human bone marrow derived MSCs [105, 106]. While upstream signaling (PTEN, p53 etc) is key to optimum induction of the autophagic pathway, the lysosomal coordinated mTORC1 activity [107] and downstream signaling involved in autophagosome/lysosomal fusion (HDAC6, TFEB) [108, 109] also contribute to a functional autophagic pathway. We hypothesize that it may be the lysosomal turnover of autophagosomes that plays a critical role in stem cell renewal, proliferation, and aging.

## Role of autophagy in stem cell-based therapeutics

Stem cell transplantation is becoming a popular therapeutic approach to treat various central nervous system (CNS) disorders, heart failure, diabetes and osteoarthritis, cancers, and neurodegeneration along with other age related degenerative disorders, and this approach is yielding significantly promising results. One of the major barriers opposing these transplantations is poor survival of the engrafted stem cells; therefore substantial efforts have been directed towards increasing their endurance. As this involves understanding the mechanisms that regulate survival and death of these stem cells, elucidating the role of autophagy in stem cells will be critical in prolonging the survival of engrafted stem cells. A very recent in vitro study has shown that survival of MSCs post transplantation in damaged myocardium can be enhanced by drugs like atorvastatin, which activates autophagy via the AMPK/mTOR pathway and helps cells survive through post graft ischemic environment of hypoxia and serum deprivation and evade apoptosis [110]. Autophagy’s role here as energy provider clearly is an attractive strategy towards its application during stem cell therapies in regenerative medicine and needs to be further explored. This also points towards the need for further research on drugs which effect autophagy pathway (e.g., statins and chloroquine) that are currently in use or could be envisaged to be used during stem cell transplantation, i.e., drug repositioning.

The use of multipotent neural stem cells in ASC-based therapies to target neurodegenerative diseases is becoming increasingly popular [111, 112]. Hippocampal neural stem cells (HCN) can differentiate into neurons, astrocytes, or oligodendrocytes in vitro and following grafting in adult human brain. Transmission electron microscopy analysis of insulin deprived dying HCN revealed autophagic vacuoles containing cytoplasmic content, while accumulation of beclin1 and LC3II was also detected. An increase in autophagic flux was further confirmed with lysosomal protease inhibitors E64D/PepA. Suppressing autophagy using Atg7-siRNA in insulin-deficient HCN cells inhibited cell death and inducing it with rapamycin accelerated cell death [113]. However, this is in contrast with most in vivo studies indicating that autophagy is a cytoprotective mechanism, with autophagic vacuoles formation merely accompanying cell death [114], therefore this study may have to be repeated in more physiological settings.

Rapamycin-induced autophagy also promotes radiosensitivity and differentiation of glioma initiating cells [115, 116] and a combination of radiotherapy and rapamycin is recommended as a potential therapeutic strategy to enhance current treatments for patients with glioblastoma, an aggressive brain tumor. While glioma stem/progenitor cells (GSPCs) have low autophagic activity when compared to neural stem/progenitor cells (NSPCs) [116], low autophagic activity in other cancer stem cells has also been reported [117]. Fetal calf serum or rapamycin promoted differentiation of GSPCs, which was accompanied by increased autophagy, while the autophagy inhibitors 3MA and BafA prevented serum-induced differentiation. Many studies have shown that the loss or mutation of PTEN occurs during malignant transformation of stem cell progenitors [118–120] and reduced PTEN expression is associated with the capability for self-renewal in cancer stem cells [121] and a loss of autophagic activity [122]. Overexpression of PTEN promoted autophagy in GSPCs. These results suggest that targeting autophagy through PTEN might have potential therapeutic value in glioma cancers.

Finally, questions arise regarding the role of autophagy in stem cell therapeutics: could autophagy, a highly networked program with the potential for clearing away specific sets of macromolecules or as an energy provider under hypoxic and serum deprived conditions, be increased for therapeutic purposes, or must autophagy/cellular metabolism be kept in check? Future research attention should be focused towards defining this balancing act, with the possibility of therapeutic genetic modulation of this pathway (master switches like GATA-1 ([123], TFEB and FOXO3), or use of pharmacological inhibitors to achieve similar results.

Nonetheless, another imperative question is whether autophagic-specific factors influence the ‘fate-direction’ of these multipotent stem cells towards a specific cell lineage? The process of reprogramming somatic cells to become induced pluripotent stem cells (iPSCs) is one such example. Pluripotency potential and genomic integrity or stability are the two most important criteria for achieving successful reprogramming of somatic cells and studies have shown that ROS is one of the fundamental triggers in reprogramming barriers like senescence [124]. In fact, rapamycin augments the reprogramming of somatic cells to iPSCs and maintains the cells in a quiescent state rather than pushing them to senescence as the cells retain the capacity to resume proliferation, in response to reprogramming-induced senescence and cell cycle arrest [125]. Also data is now available to link autophagy promoting molecules to differentiation of hESCs, for example osteoblastic differentiation of hESCs is promoted by rapamycin [24, 126]. Of course, it needs further research to ultimately pin down this process, eventually enabling the search for targeted small molecules to induce and inhibit autophagy.
